# Antivirulence Therapy for Animal Production: Filling an Arsenal with Novel Weapons for Sustainable Disease Control

**DOI:** 10.1371/journal.ppat.1003603

**Published:** 2013-10-10

**Authors:** Tom Defoirdt

**Affiliations:** 1 Laboratory of Aquaculture and Artemia Reference Center, Ghent University, Ghent, Belgium; 2 Laboratory of Microbial Ecology and Technology, Ghent University, Ghent, Belgium; University of North Carolina at Chapel Hill School of Medicine, United States of America

## Antivirulence Therapy as an Alternative to Antibiotics

Antibiotics are still critically important as a first line therapy for the treatment of various bacterial infections in the clinic. In addition to their use in human medicine, these compounds have also been used for decades in animal production, for both growth promotion and veterinary purposes [1], [2]. Because of the development and spread of antibiotic resistance, there is a growing awareness that antibiotics should be used with more care [3], and as a consequence, the development of alternative methods to control pathogenic bacteria in animal production will be important to ensure good productivity in the future.

**Figure 1:**
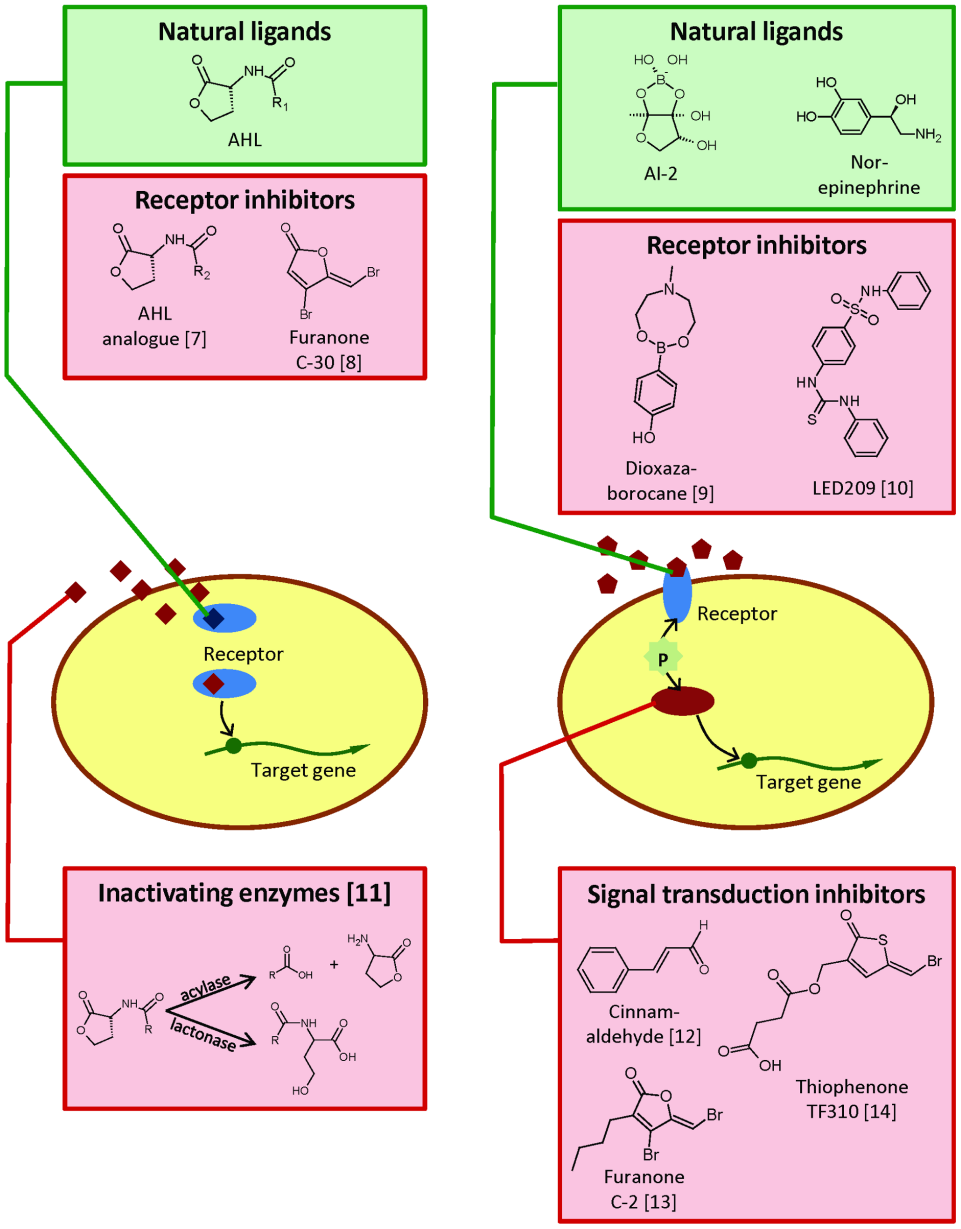
Simplified schematic representation of virulence regulatory systems based on detection of signal molecules in animal pathogenic bacteria. These include **(left)** quorum sensing based on acylhomoserine lactones (AHL) and **(right)** quorum sensing in vibrios and catecholamine stress hormone sensing. For each type of system, examples of natural ligands, receptor inhibitors, and other inhibiting agents are shown. Dioxazaborocane is an inhibitor of AI-2 sensing in *V. harveyi* and LED209 is an inhibitor of catecholamine sensing in *E. coli*. The signal transduction inhibitors are inhibitors of quorum sensing signal transduction in vibrios.

Infection of both terrestrial and aquatic animals by bacterial pathogens requires the production of different virulence factors, i.e. gene products that allow the pathogenic bacteria to enter and damage the host. Major virulence factors include gene products involved in motility, adhesion, host tissue degradation, iron acquisition, secretion of toxins, and protection from host defense [4]. As virulence factors are required for infection, preventing pathogens from producing them constitutes an interesting alternative strategy for disease control, a strategy that has been termed antivirulence therapy [5]. Antivirulence therapy is based on a thorough understanding of the mechanisms by which bacterial pathogens cause disease. In this respect, studies aimed at understanding how bacteria cause disease have identified (and will probably continue to do so) targets for therapeutics with completely novel modes of action. Inhibitors of specific virulence factors, such as secretion systems, have been reported in literature [6]. However, considerably more research effort is being directed towards interference with regulatory mechanisms that control the expression of (multiple) virulence factors, such as bacterial cell-to-cell communication (quorum sensing) and host-pathogen signalling (**Fig. 1**). The following paragraphs will focus on interference with these mechanisms as a novel strategy to control animal pathogens, using *Escherichia coli* and *Salmonella* spp. as examples of pathogens for terrestrial animals, and *Aeromonas* spp. and *Vibrio* spp. as examples of aquatic pathogens.

## Interfering with Bacterial Cell-to-Cell Communication in Animal Pathogens

Quorum sensing, or bacterial cell-to-cell communication, is a mechanism of gene regulation in which bacteria coordinate the expression of certain genes in response to the presence of small signal molecules. This regulatory mechanism has been shown to control virulence gene expression in many different pathogens, and a wide range of molecules (both of natural and synthetic origin) able to interfere with quorum sensing systems have been reported (for a recent review see [15]). Quorum sensing has been documented to be required for full virulence of *Aeromonas* spp. and vibrios towards different aquatic hosts, including fish and crustaceans [16]–[18]; moreover, different quorum sensing-disrupting agents have been proven effective in controlling disease. Effective compounds include cinnamaldehyde, brominated furanones and brominated thiophenones [14], antagonistic acylhomoserine lactones [7], and signal molecule-degrading enzymes [11]. Virulence-related phenotypes (including motility and adhesion) of *E. coli* and *Salmonella* spp. have also been reported to be controlled by quorum sensing molecules [19], [20], and the signal molecule indole has been shown to affect killing of the nematode *C. elegans* [21]. However, to the best of my knowledge, no reports have been published thus far mentioning the successful use of inhibitors of these types of bacterial cell-to-cell communication to protect terrestrial farmed animals from disease caused by these pathogens. The evaluation of these kind of compounds in terrestrial animals should be rather straightforward, as many inhibitors have been isolated and/or synthesised [15]. Although the peptide quorum sensing systems of Gram-positive bacteria thus far have received much less attention than acylhomoserine lactone systems in Gram-negative bacteria, some inhibitors of these systems have been documented as well (e.g. cyclic peptide inhibitors of quorum sensing in staphylococci [22]), and these kind of compounds might also prove effective in controlling animal diseases caused by Gram-positive pathogens.

## Interfering with Host-Pathogen Signalling in Animal Pathogens

In addition to bacterial signals, *E. coli* and *Salmonella* spp. can also sense and respond to host cues such as the catecholamine stress hormones adrenaline and noradrenaline. These hormones are an integral part of the acute “fight or flight” stress response in animals and are conserved among vertebrates and invertebrates. Catecholamines can facilitate the removal of iron from host iron-binding proteins, thereby making it available to the bacteria and increasing their growth under iron-limited conditions [23]. In addition to their growth-stimulatory effect, catecholamines also increase virulence gene expression of pathogenic bacteria. In different pathogenic *E. coli* strains, the compounds have been reported to affect the production of virulence-related phenotypes such as motility and type III secretion [24], Shiga toxin expression [25], and expression of pilus and fimbrial adhesins [26]. In *Salmonella* spp., they have been reported to affect motility [27], hemolysin production [28], type III secretion [10], and intestinal colonization in chicks, pigs, and calves [29], [30]. Different bacterial adrenergic sensors have recently been described (with the best-described one being QseC), showing different susceptibilities to blocking with eukaryotic α- and β- adrenergic receptors, respectively [31], [32]. An inhibitor of bacterial catecholamine sensing, LED209, has also been described [10]. It needs to be noted that (at least in *Salmonella* spp.) different research groups have reported conflicting effects of catecholamines, which may reflect differences in host species, bacterial strains, routes of infection, and nature of mutations [23], [31], [32]. Interestingly, vibrios and *Aeromonas* spp. also respond to catecholamines, and QseC homologues have been reported in these bacteria as well [33].

## Advantages of this Strategy

When compared to the use of antibiotics, a major advantage of antivirulence therapy is that there will be less interference with non-target organisms (i.e. the commensal microbiota), as it specifically targets virulence gene expression or virulence gene regulation; in the latter case there might be some interference with regulatory mechanisms in non-target organisms. Moreover, because such a strategy will pose selective pressure only under conditions in which the virulence genes are required, the tendency towards resistance development and spread will probably also be lower (though not absent) [34]. It should be noted, however, that some of the resistance mechanisms that bacteria have acquired during exposure to antibiotics can also render them resistant to antivirulence agents. This was recently demonstrated in *Pseudomonas aeruginosa*, in which clinical isolates showing an increased expression of a multidrug efflux pump were also resistant to a quorum sensing-disrupting brominated furanone [35]. A major advantage of targeting the regulatory mechanisms described above is that agents can be used that do not need to enter the cells to exert their activity (e.g., signal molecule-degrading enzymes or compounds that interfere with cell surface receptors). Consequently, pre-existing nonspecific resistance mechanisms (e.g. multidrug efflux pumps and decreased cell membrane permeability) will not alter the effectiveness of such agents.

## Conclusion

It is of significant interest to further develop antivirulence therapy as a novel biocontrol strategy for animal production. Further research is needed to document the impact of such a strategy in different host-pathogen settings and to continue the quest for novel antivirulence agents, i.e. inhibitors of either natural or synthetic origin, or microorganisms able to interfere with virulence (regulatory) mechanisms.
